# Caspase-Mediated Apoptosis in Sensory Neurons of Cultured Dorsal Root Ganglia in Adult Mouse

**Published:** 2013-08-24

**Authors:** Hamid Reza Momeni, Malek Soleimani Mehranjani, Mohammad Ali Shariatzadeh, Mahnaz Haddadi

**Affiliations:** Department of Biology Faculty of Science, Arak University, Arak, Iran

**Keywords:** Apoptosis, Caspase, Dorsal Root Ganglia, Sensory Neurons

## Abstract

**Objective:**

Sensory neurons in dorsal root ganglia (DRG) undergo apoptosis after peripheral nerve injury. The aim of this study was to investigate sensory neuron death and the mechanism involved in the death of these neurons in cultured DRG.

**Materials and Methods:**

In this experimental study, L5 DRG from adult mouse were dissected and incubated in culture medium for 24, 48, 72 and 96 hours. Freshly dissected and cultured DRG were then fixed and sectioned using a cryostat. Morphological and biochemical features of apoptosis were investigated using fluorescent staining (Propidium iodide and Hoechst 33342) and the terminal Deoxynucleotide transferase dUTP nick end labeling (TUNEL) method respectively. To study the role of caspases, general caspase inhibitor (Z-VAD.fmk, 100 μM) and immunohistochemistry for activated caspase-3 were used.

**Results:**

After 24, 48, 72 and 96 hours in culture, sensory neurons not only displayed morphological features of apoptosis but also they appeared TUNEL positive. The application of Z-VAD.fmk inhibited apoptosis in these neurons over the same time period. In addition, intense activated caspase-3 immunoreactivity was found both in the cytoplasm and the nuclei of these neurons after 24 and 48 hours.

**Conclusion:**

Results of the present study show caspase-dependent apoptosis in the sensory neurons of cultured DRG from adult mouse.

## Introduction

Organ culture allows for the preservation of organ structural properties with realistic cellcell interaction as observed *in vivo*. In this context, the culture of dorsal root ganglia (DRG) has become a useful tool for studying axonal out growth (1), cell survival (2) and cell death (3). Although cultured DRG offers clear advantages for* in vitro* studies, the organ must be removed from the animal in the process of which multiple axotomies are performed on its sensory neurons. The sensory neurons that undergo nerve axotomy and consequently target deprivation initiate a cascade of events which are involved in neuronal survival and therefore axonal regeneration (1). Although adult sensory neurons are much more resistant to axotomy insult, around one third or more of the axotomized neurons display both morphological and biochemical features of cell death (4). In such cultures, the first prerequisite step for axonal regeneration is dependent on the survival of the axotomized neurons. Therefore, the study of mechanisms responsible for cell death in these neurons could be a useful strategy for promoting neuronal survival and subsequent axonal regeneration.

Several previous studies have demonstrated cell death in the sensory neurons after sciatic nerve transection in neonatal rats (5), adult rats (4, 6) and adult mice (7, 8). In all cases where the sciatic nerve has been subjected to an injury, apoptosis seems to be the dominant way by which sensory neurons die. In spite of several studies in which apoptosis has been shown to be triggered in sensory neurons after peripheral nerve injury, little research has reported cell death in adult cultured DRG not subjected to an insult. Although Lindwall and co-workers (3) demonstrated apoptosis as well as survival in sensory neurons in cultured DRG from new born rats, they only focused on mechanisms involved in survival and regeneration of the sensory neurons and did not explore mechanisms responsible for apoptotic neurons. Therefore, the present study was performed to examine sensory neuron death and the mechanisms which mediate the death of these neurons in adult cultured DRG.

## Materials and Methods

### Animals and preparation of DRG

In this experimental study adult female Balb/c mice (23-25 g, 5-6 weeks old) were used. The animals were purchased from the Pasteur Institute, Tehran, Iran. The animals were housed in plastic cages with a 12 hour light/ dark cycle, (21 ± 2˚C) with water and food ad libitum. The experiments were approved by the local Ethics Committee at Arak University. The animals were deeply anesthetized by an intraperitoneal injection of sodium pentobarbital (60 mg/kg) and subsequently killed by heart puncture. L5 DRG (n=18 from 9 different animals) were rapidly dissected under aseptic conditions, placed in ice cold phosphate buffered saline (PBS, pH=7.4) and cultured in a medium composed of a mixture of 50% minimum essential medium, 25% Hanks balanced salt solution, 25% horse serum, 25 mM N-2-hydroxyethyl piperazine-N’-2-ethanesulfonic acid (HEPES), 6 g/L glucose and 1% penicillin-streptomycin, pH=7.3- 7.4. The cultures were incubated at 37˚C in a humidified atmosphere of 5% CO_2_ in air for different periods of time.

### Fixation and sectioning

Freshly prepared (0 hour) and cultured slices were fixed in Stefanini’s fixative (2% paraformaldehyde, 0.2% picric acid in 0.1 M phosphate buffer, pH=7.2) for at least 2 hours. The fixed DRG were washed in PBS (3×5 minutes) and incubated overnight in 20% sucrose in PBS at 4˚C. The DRG were cut into 10 µm-thick sections in a cryostat. The sections were collected and mounted on Poly-L-lysine coated glass slides. The slides were kept at -20˚C until use.

### ssessment of apoptosis

A In the sensory neurons, apoptosis was assessed by fluorescent staining and the TUNEL method. To study morphological features of apoptosis, a combination of propidium iodide (PI, Sigma, USA, 10 µg/ml in PBS; 5 minutes at room temperature) and Hoechst 33342 (Sigma, USA, 5 µg/ml in PBS; 30 seconds at room temperature) was used. The cryostat sections were washed in PBS (3×5 minutes), mounted in glycerol/ PBS (1:1) and coverslipped. Photographs were taken with an Olympus camera attached to an Olympus fluorescence microscope (Olympus Optical Co Ltd, Japan). The TUNEL assay, used to investigate biochemical feature of apoptosis, was carried out according to the kit manufacturer’s protocol (Chemicon, USA). The sensory neurons were then photographed under a light microscope.

### Evaluation of the involvement of caspases

Caspase inhibitor and immunohistochemistry for activated caspase-3 were used to study the role of caspases in the apoptosis of the sensory neurons. General caspase inhibitor, N-Benzyloxycarbonyl-Val-Ala-Asp (O-Me) fluoromethyl ketone (Z-VAD.fmk, Sigma, USA, 100 μM), was dissolved in dimethylsulfoxide (DMSO) as stock solutions and stored at -20˚C. The stock solution was directly added to the medium. Controls also received a corresponding amount of DMSO. For immunohistochemistry, the cryostat sections were washed in PBS (3×5 minutes) and incubated with a 1:200 dilution of a rabbit antibody against the active form of caspase-3 (Cell Signaling and Technology, USA) in a moist chamber at 4˚C overnight. The sec-m tions were washed in PBS (3×5 minutes) and incubated with goat anti rabbit Alexa 488 (Molecular Probes, USA) labeled secondary antibody at room temperature for 1 hour. Negative controls were performed using alternative sections which were incubated without the primary antibody. The sections were washed in PBS (3×5 minutes) and counterstained with Hoechst 333425 (5 μg/ml in PBS). The sections were then washed in PBS (3×5 minutes), mounted in glycerol/ PBS solution (1:1) and coverslipped. Photographs were taken with the fluorescence microscope using the appropriate excitation and emission filters.

## Results

### Apoptosis in sensory neurons

**Fig 1:**
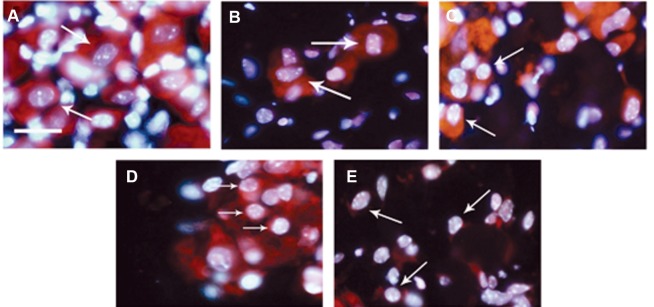
Morphological features of apoptosis in sensory neurons. Propidium iodide (red) and Hoechst 33342 (blue) staining revealed morphological changes of apoptosis in dorsal root ganglion (DRG) sensory neurons. A. normal sensory neurons with large cell body and nucleus from freshly prepared DRG (0 hour). Sensory neurons after 24 (B), 48 (C), 72 (D) and 96 hours (E) displayed cell shrinkage as well as nuclear and chromatin condensation. Scale bar: 25 µm. Arrows show sensory neurons.

**Fig 2:**
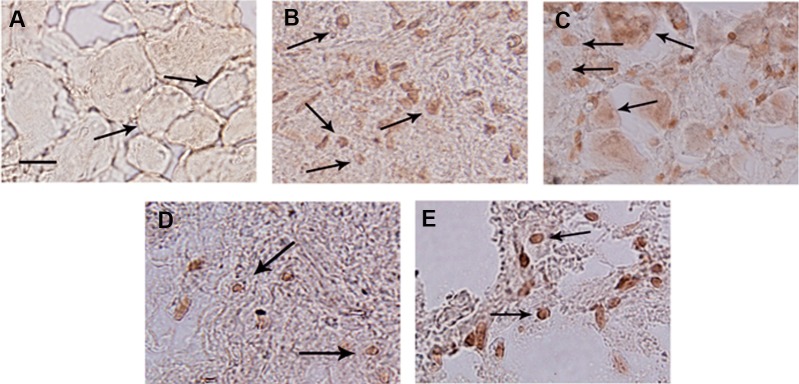
NA fragmentation during apoptosis of dorsal root ganglia (DRG) sensory neurons using TUNEL method. A. sensory neurons from freshly dissected DRG (0 hour) appeared TUNEL negative. TUNEL positive sensory neurons after 24(B), 48(C), 72(D) and 96(E) hours in culture. Scale bar: 25 µm. Arrows show sensory neurons.

Sensory neurons from freshly dissected DRG (0 hour) revealed a large cell body, large nucleus and the expected distribution of nuclear material with no apoptotic signs (Fig 1A). After 24 hours in culture, some of the sensory neurons displayed cell shrinkage as well as nuclear and chromatin condensation (Fig 1B ). After 48 hours (Fig 1C ), 72 hours (Fig 1D ), and 96 hours (Fig 1E ) the cytoplasmic and nuclear changes were extensive. The morphological results were further characterized using the TUNEL method (Fig 2). The sensory neurons from freshly prepared DRG showed no TUNEL positive cells (Fig 2A). In contrast, a considerable number of TUNEL positive nuclei were detected in the neurons from DRG cultured for 24, 48, 72 and 96 hours (Figs 2B, C, D , and E, respectively).

### Caspase-dependent apoptosis in sensory neurons

**Fig 3:**
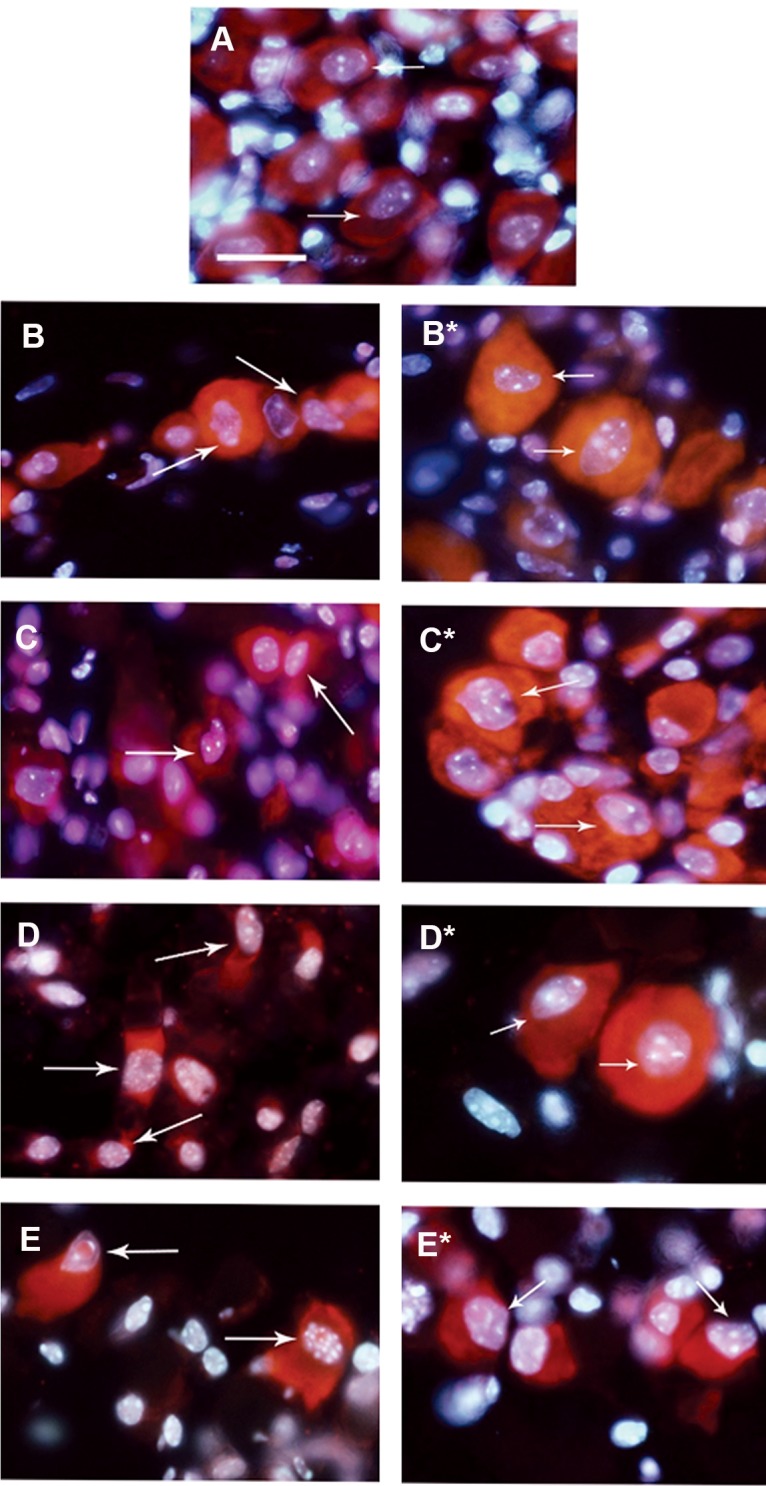
Caspase-dependent apoptosis in dorsal root ganglia (DRG) sensory neurons. A: Sensory neurons from freshly prepared DRG (0 hour). B, C, D, and E: Sensory neurons from DRG cultured for 24, 48, 72 and 96 hours respectively (controls). B', C', D' and E': Sensory neurons from cultured DRG in the presence of general caspase inhibitor (Z-VAD.fmk, 100 µM) after 24, 48, 72 and 96 hours. Scale bar: 25 µm. Arrows show sensory neurons.

The application of general caspase inhibitor (ZVAD.fmk, 100 μM) for 24, 48, 72 and 96 hours (Figs 3A, B, C, D, and E respectively) considerably inhibited apoptosis in the treated sensory neurons compared to the controls (Fig 3B', C', D', and E' respectively).

**Fig 4:**
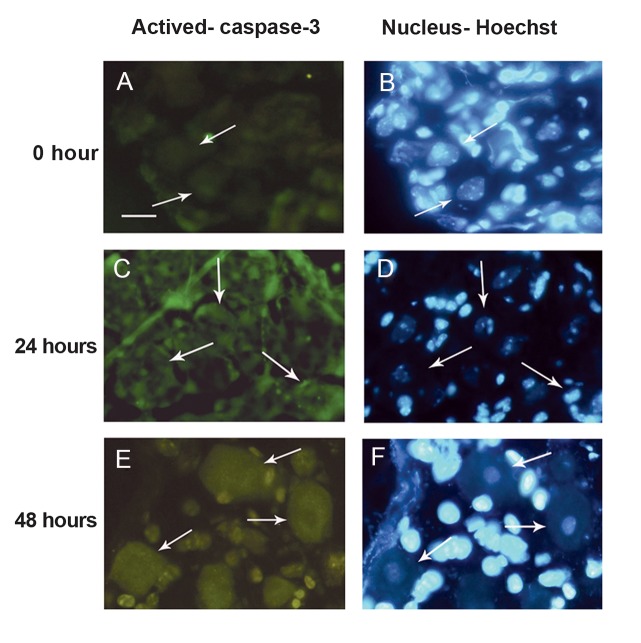
: Immunolocalization of activated caspase-3 antibody in dorsal root ganglia (DRG) sensory neurons. Sensory neurons were stained with activated caspase-3 antibody (green) and counterstained with Hoechst 33342 (blue). (A-B).Weak activated caspase-3 immunoreactivity in sensory neurons from DRG at 0 hour with no sign of apoptotis. Sensory neurons from DRG cultured for 24 hours (C-D) and 48 hours (E-F) displayed intense activated caspase-3 immunoreactivity both in the nucleus and the cytoplasm where the nuclei showed apoptotic features. Scale bar: 25 µm. Arrows show sensory neurons.

The active form of caspase-3 antibody was used to further identify the role of caspase in the sensory neurons. Sensory neurons from freshly dissected DRG showed weak immunoreactivity for activated caspase-3 in the cytoplasm (Fig 4A, B). After 24 hours (Fig 4C,D) and 48 hours (Fig 4E, F) intense activated caspase-3 immunoreactivity was found both in the cytoplasm and the nucleus of the sensory neurons where such neurons displayed nuclear and chromatin condensation. Similar immunoreactivity was also observed in the sensory neurons from DRG cultured for 72 and 96 hours (data not shown).

## Discussion

In this study we examined the involvement of caspase in the apoptosis of adult sensory neurons. The sensory neurons in cultured DRG displayed cell shrinkage as well as nuclear and chromatin condensation. Such changes have been documented as morphological features of apoptosis (9). Since apoptosis could not be confirmed by just analyzing the morphological changes in the nucleus and chromatin, we also used the TUNEL method which facilitates the visualization of DNA fragmentation in several apoptotic cell models (10-12). However, this method is limited because it can detect both apoptotic and non-apoptotic cells, unless it is accompanied by other techniques (13). Altogether, observed changes in the sensory neurons provided evidence that apoptosis could be one of the reasons by which neurons perish in culture. The question of whether the present results are a reflection of the cell death that occurs in response to organ culture or whether they reflect events that can also occur *in vivo* is interesting. It has been demonstrated that, as a result of peripheral nerve injury, apoptosis is induced in DRG sensory neurons (5, 7). Therefore we can speculate that apoptosis of sensory neurons is in response to the axotomy of sensory neurons.

The molecular mechanisms behind the apoptosis of sensory neurons in cultured DRG have not been elucidated. Since apoptosis occurs through two different mechanisms, caspase dependent and caspase independent (14, 15), in the present study one possibility might be the activation of caspases which play a critical role in the execution of apoptosis (16). If caspases were a possible mechanism for apoptosis of the sensory neurons, the inhibition of caspases should prevent apoptosis in these neurons. Interestingly, the caspase inhibitor, Z-VAD. fmk, was able to provide effective protection in the sensory neurons. In this context, several reports suggest that Z-VAD.fmk attenuates caspase-dependent apoptosis in neurons (11, 17). Caspase-3 is one of the most important effector caspases which has been expressed in several kinds of apoptotic neurons (11, 17). We therefore hypothesize that caspase-3 might be activated in the apoptotic sensory neurons. Using an antibody specific to activated caspase-3, we found intense immunorectivity for the activation of caspase-3 both in the cytoplasm and the nuclei of the apoptotic neurons. Our results showed that the induction of apoptosis in the sensory neurons, as revealed by fluorescent staining and the TUNEL assay, was increased in a time-dependent manner. In addition, the time course of apoptosis was consistent with the time course of caspase-3 activation in these neurons. Taken together, our results suggest that caspase-3 could be involved in the apoptosis of sensory neurons following DRG culturing. The finding that weak activated caspase-3 immunoreactivity was observed within the cytoplasm of sensory neurons in freshly prepared DRG may suggest the presence of activated caspase-3 under physiological conditions. During DRG culturing, the localization of activated caspase-3 in the nuclei of sensory neurons was interesting and could be due to the translocation of this protease from the cytoplasm to the nucleus. Such transloction has been demonstrated by Scholz et al. (11) in DRG sensory neurons after peripheral nerve injury. The appearance of activated caspase-3 in the sensory neurons may explain cytoplasmic and nuclear apoptotic changes in these neurons. In caspase-dependent apoptosis, activated caspases cleave cytoplasmic proteins e.g. actin and fodrin (18) as well as nuclear substrates such as lamins (19), leading to the disintegration of cellular structure. The activation of endonucleases such as caspase activated DNase (CAD) could be another mechanism for nuclear apoptotic changes in the sensory neurons. Activated caspase-3 cleaves ICAD (inhibitor of CAD), to release CAD; the active form of this protease. Once activated, it translocates into the nucleus to induce chromatin condensation and DNA fragmentation (20, 21). Therefore, it is reasonable to assume that the cytoplasmic and nuclear apoptotic changes in the sensory neurons were due to the activity of protease(s) and nuclease(s).

## Conclusion

The immunolocalization of activated caspase-3 in apoptotic sensory neurons as well as the possibility that the caspase inhibitor could delay apoptosis in the neurons suggest that caspases, in particular caspase-3, might be involved in the apoptosis of adult sensory neurons. 
